# Low-dose CT from myocardial perfusion SPECT/CT allows the detection of anemia in preoperative patients

**DOI:** 10.1007/s12350-021-02899-x

**Published:** 2022-02-17

**Authors:** Antonio G. Gennari, Hannes Grünig, Dominik C. Benz, Stephan Skawran, Alexander Maurer, Ahmad M. A. Abukwaik, Alexia Rossi, Catherine Gebhard, Ronny R. Buechel, Michael Messerli

**Affiliations:** 1 Department of Nuclear Medicine, University Hospital Zurich/University of Zurich, Ramistrasse 100, 8091 Zurich, Switzerland; 2 University of Zurich, Zurich, Switzerland; 3 Center for Molecular Cardiology, University of Zurich, Zurich, Switzerland; 4 Maastricht UMC+, Heart and Vascular Center, Maastricht, The Netherlands

**Keywords:** Anemia, Hematocrit, Hemoglobin, Tomography, X-ray computed, Tomography, Emission-computed, Single-photon

## Abstract

**Background:**

To assess whether low-dose CT for attenuation correction of myocardial perfusion single-photon emission computed tomography (SPECT) allows for identification of anemic patients and grading anemia severity.

**Methods and Results:**

Patients who underwent a preoperative blood-test and low-dose CT scan, as a part of a cardiac SPECT exam, between 01 January 2015 and 31 December 2017 were enrolled in this retrospective study. Hemoglobin (Hb) levels and hematocrit were derived from clinical records. CT images were visually assessed (qualitative analysis) for the detection of inter-ventricular septum sign (IVSS) and aortic rim sign (ARS) and quantitative analysis were performed. The diagnostic accuracy for detecting anemia was compared using Hb values as the standard of reference. A total of 229 patients were included (110 with anemia; 57 mild; 46 moderate; 7 severe). The AUC of IVSS and ARS were 0.830 and 0.669, respectively (p<0.0001). The quantitative analysis outperformed ARS and IVSS; (AUC of 0.893, p=0.29). The optimal anemia cut-off using Youden index was 4.5 HU.

**Conclusion:**

Quantitative analysis derived from low-dose CT images, as a part of cardiac SPECT exams, have a diagnostic accuracy similar to that of hematocrit for the detection of anemia and may allow discriminating different anemia severities.

**Graphical abstract:**

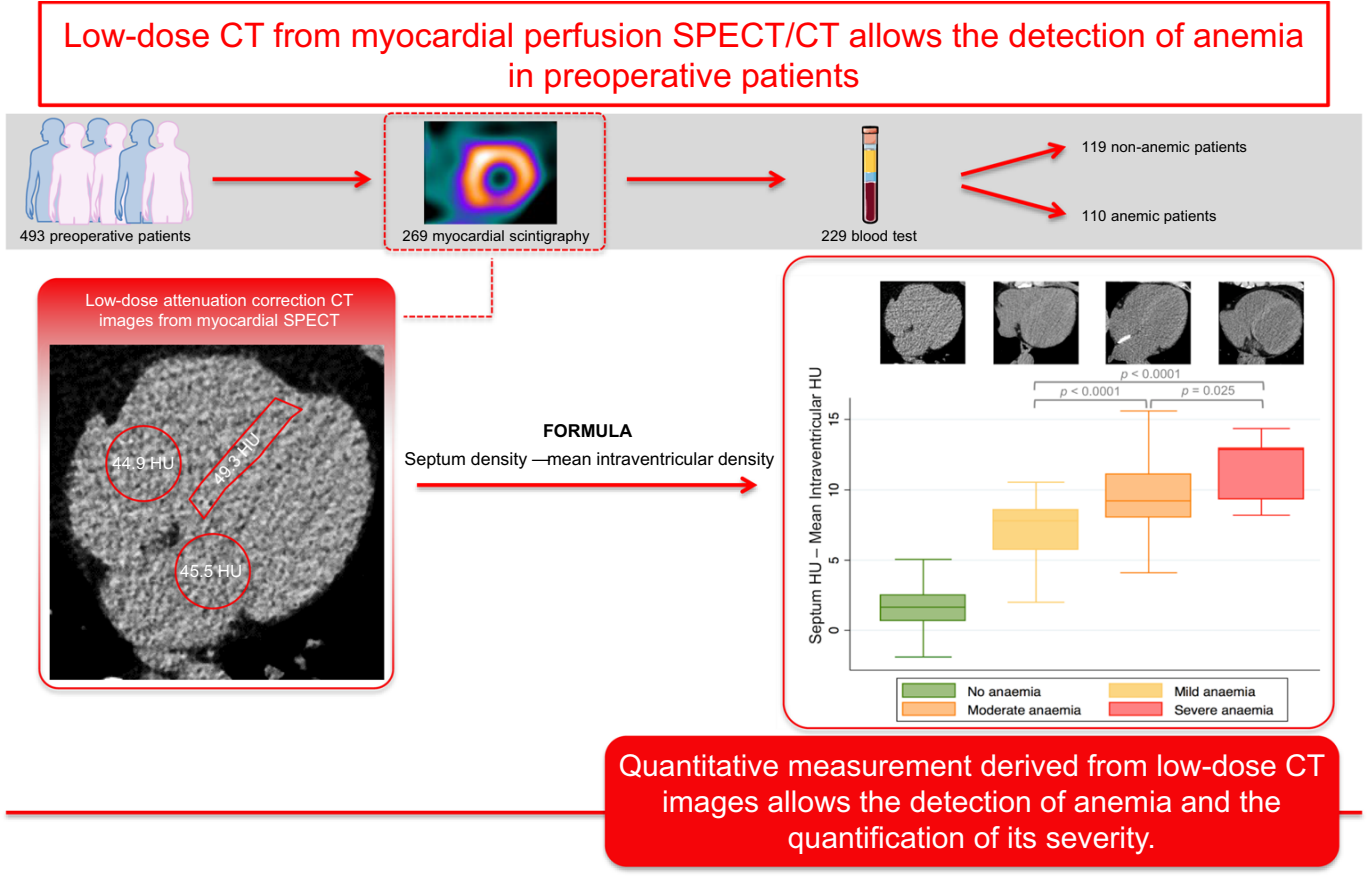

**Supplementary Information:**

The online version contains supplementary material available at 10.1007/s12350-021-02899-x.

## Introduction

Anemia is defined as a mismatch between the circulating concentration of red blood cells and the physiological needs. According to the World Health Organization (WHO) 1.6 billion people worldwide are affected by this condition.^1^ Several factors contribute to anemia development, among which iron deficiency is the most frequent cause.^2^ Hemoglobin (Hb) concentration and hematocrit are the laboratory parameters currently used to diagnose and follow-up anemia. Hb measurement is relatively inexpensive, and it is considered the most reliable indicator of anemia by the WHO.^1,2^ Although hematocrit (i.e., the volume percentage of red blood cells in the total blood volume) is a less accurate anemia marker,^3^ it is still used in several countries, and conversion formulas between hematocrit and Hb are available.^3^

Many studies have found a direct relationship between anemia and other comorbidities,^4–7^ as well as to higher mortality rates.^7–9^ Moreover, in preoperative patients, anemia has been linked to poorer surgical outcome rates and to a higher need for red blood cells transfusion.^10–13^ The latter directly increases post-surgical hospital length of stay, morbidity, and mortality rates.^14,15^ Also, preoperative anemia correction through oral iron supplementation takes about 4 to 6 weeks, delaying scheduled surgical treatments.

Myocardial perfusion single-photon emission computed tomography (SPECT) is frequently used to preoperatively stratify cardiovascular risk in patients with elevated cardiovascular risk and poor functional capacity.^16^ As part of the examination, low-dose unenhanced computed tomography (CT) images serve for attenuation correction and for coronary calcium burden estimation. Unenhanced CT already proved its ability to detect anemia.^17–20^ Among others, the *aortic rim sign* (ARS) and the *inter-ventricular septum sign* (IVSS), differentiating high-density parenchymal structures from low-density intravascular blood, were associated to the highest detection rates of anemia.^19^ However, unenhanced chest CT are not routinely in preoperative patients. Also, to the best of our knowledge, studies on anemia detection using SPECT-derived low-dose CT images are lacking.

Therefore, in our study, we aimed at evaluating the diagnostic accuracy of preoperative low-dose CT images from myocardial SPECT/CT perfusion studies for the detection of anemia, comparing qualitative and quantitative analysis.

## Material and Methods

### Study Population

**Figure 1:**
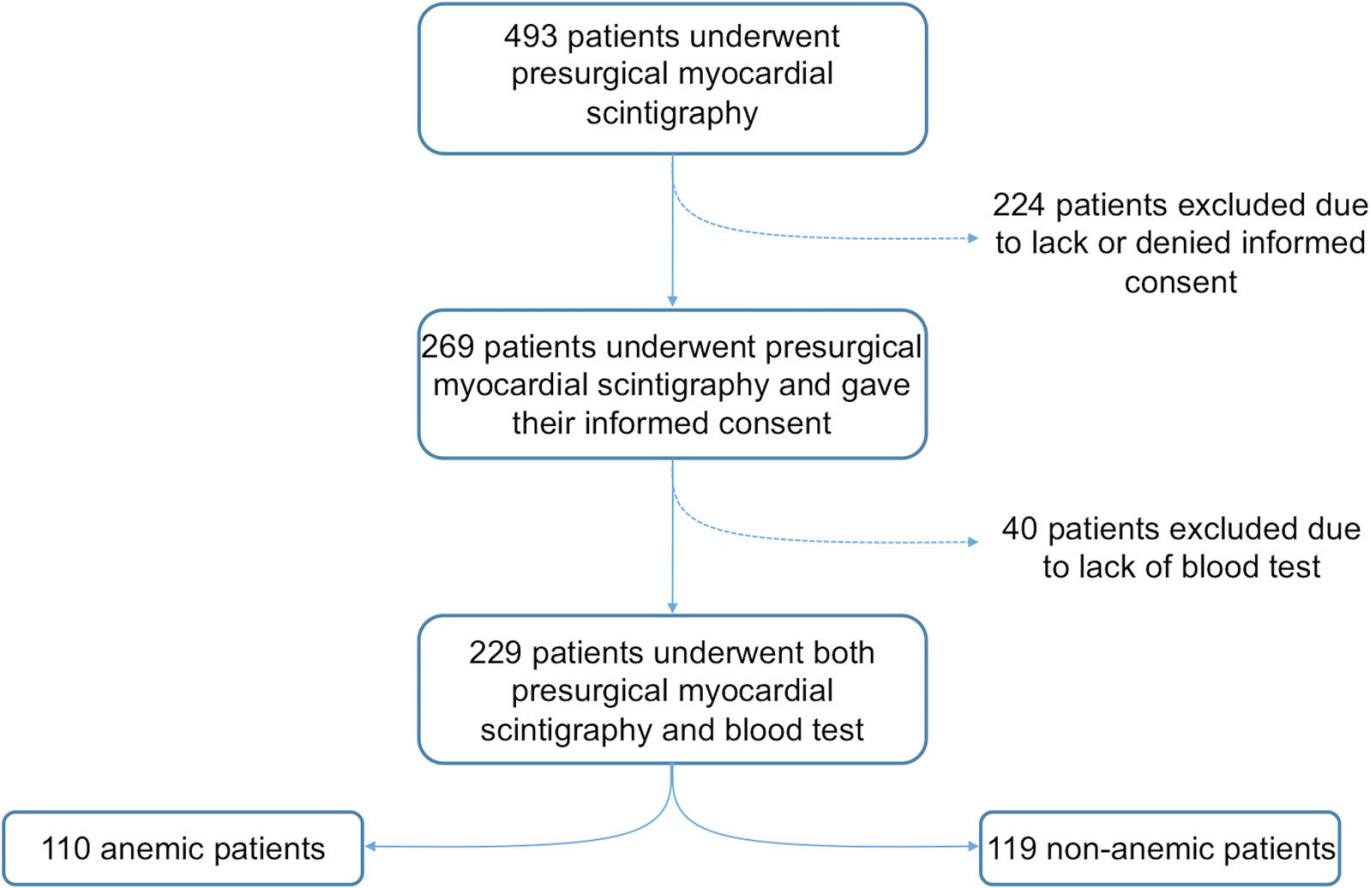
Flow diagram of the study group

This is a cross-sectional, single-center, retrospective study. We included all patients who underwent a preoperative myocardial perfusion SPECT/CT, between 1st of January 2015 and 31st of December 2017, at the Nuclear Medicine Department of the University Hospital of Zurich, for whom Hb and hematocrit values were available (Figure 1). Exclusion criteria were as follows: *(1)* age < 18 years old; *(2)* systemic syndromes affecting myocardium density, such as vasculitis, glycogen accumulation, or hepatic hemochromatosis; *(3)* contrast media administration within 2 days before SPECT/CT acquisition; *(4)* image artifacts precluding accurate cardiac evaluation; *(5)* blood-test acquired after surgery. Medical records were used to derive patients’ clinical data. The surgical procedures were categorized using a simplified per-organ, per-apparatus, or per-procedure approach as follows: liver surgery/transplantation, kidney surgery/transplantation, lung surgery/transplantation, back surgery, abdominal surgery, vascular surgery, orthopedic surgery, bariatric surgery, or others. The “abdominal surgery” category comprised all procedures involving the gastro-enteric tract. The group named “others” included surgical procedures not identified by the other groups (e.g., neurosurgical procedures, pancreatic surgery, cystectomy, prostatectomy). The institutional review board approved the study; written informed consent for the use of medical data were obtained from all patients.

### Definition of Study Endpoint

Anemia was defined as Hb levels < 134 g/L for men and < 117 g/L for women, according to the reference values routinely used at the University Hospital of Zurich. Patients were divided into four groups according to Hb values: non-anemic, mild anemic, moderate anemic, and severe anemic. Moderate or severe anemia were classified according to WHO cut-offs: 90 < Hb ≤ 60 g/L, and < 60 g/L, respectively.^2^

### Hematocrit Definition

Clinical hematocrit cut-offs routinely used at University Hospital of Zurich were adopted to discriminate between non-anemic and anemic patients (< 40% for men and < 35% for women).

### CT Image Acquisition

Myocardial perfusion images were acquired using two different SPECT scanners: Discovery NM 530 and Infinia, both from GE Healthcare, Waukesha, Wis.

CT-based attenuation correction maps of the chest were generated based on non-contrast enhanced, low-dose, CT images. All patients were scanned with a latest-generation 256-slice CT scanner (Revolution CT; GE Healthcare, Waukesha, Wis), using a single-beat acquisition during inspiratory breath-hold. Images were acquired using prospective electrocardiography triggering at 75% of the R–R interval. The following scanner parameters were adopted: tube current 200 milliamperes, tube voltage 120 kVp, 256 × 0.625 mm collimation, 12 to 16 cm *z*-axis coverage, 512 × 512 matrix, 25 cm field of view, and gantry rotation time of 280 msec. Subsequently, images were reconstructed with a slice thickness of 2.5 mm and an increment of 2.5 mm.

### CT Image Analysis

A board-certified radiologist (A.G.G., with 7 years of experience in cardio-thoracic imaging), blinded to patients’ clinical data, analyzed the CT images. All readings were performed on a Picture Archiving and Communication System (PACS)-integrated station (24-inch display, 1920 × 1080 resolution). The reader was free to use processing tools such as image scrolling, windowing, gradation adjustment, and magnification. A three-step approach was used for image analysis.

1. *Detection of anemia-qualitative analysis*. Images were visually inspected to detect CT findings of anemia (ARS, IVSS and ARS+IVSS) using both the standard mediastinum window settings (window width/window level, 30/350 HU) and an adjusted window settings (window width/window level, 50/130 HU). ARS and IVSS were defined as a hyperdense aortic wall or inter-ventricular (IV) septum compared to the blood pool.^18^ The reader scored his confidence in detecting these findings, using a 4-point scale as follows: (0) “confident on the absence”, (1) “doubtful on the absence”, (2) “doubtful on the presence”, and (3) “confident on the presence”, respectively. ARS and IVSS absence (scores 0 to 1) or presence (scores 2 to 3), and certainty rates were calculated.
2. *Detection of anemia-quantitative analysis*. Left (LV) and right (RV) intra-ventricular densities, as well as the density of the IV septum, were measured. Two circular regions of interest (ROIs) of approximately 400 mm^2^ were drawn within the LV and RV, at the level of mitral and tricuspid valve, respectively. ROIs location was selected avoiding myocardium structures (ex. papillary muscles, IV septum, etc.). To evaluated myocardium density a free-hand, cigar-shaped ROI was drawn as follows: on the IV septum, whenever visible, leaving a margin of approximately 1 mm between the IV and the ventricular chambers, or connecting the cardiac crux to incisura apicis cordis, in all other cases. The henceforth defined as quantitative analysis was calculated averaging LV and RV ROIs density and, then, subtracting it from that of IV septum as previously proposed by Zhou et al.^20^
3. *Calcified plaque burden of ascending aorta*. Aortic wall calcifications may hamper the detection of ARS. Therefore, the presence of calcified plaques was evaluated in the visible portion of the ascending aorta and scored as follows: (0) absence of calcific plaque, (1) spotty calcifications, (2) calcified plaque involving more than 10% of ascending aorta circumference.

### Intra- and Inter-reader Agreement

After two months, the images of fifty randomly selected patients were re-evaluated to assess intra-reader agreement. Further, the same cohort of patients was analyzed by a second reader (S.S. with 5 years of experience in cardio-thoracic imaging), to assess inter-reader agreement.

### Statistical Analysis

Statistical analysis was performed using commercially available software (STATA, version 14). Results were reported accordingly to Standard for Reporting of Diagnostic Accuracy criteria.^21^ Data normality was checked using the Shapiro–Wilk test. Continuous variables were presented as mean ± SD and compared using the unpaired *t* test. Non-normally distributed variables were presented as median (interquartile range, IQR) compared using the Mann–Whitney test. Finally, categorical variables were presented as frequencies and percentages. Correlation between variables was calculated using Pearson’s (*r*), or Spearman’s correlation coefficient (*r*_s_), as appropriate.

The diagnostic performances of hematocrit, qualitative and quantitative CT parameter in the detection of anemia were determined against the reference standard and expressed as sensitivity, specificity, positive predictive value (PPV) and negative predictive value (NPV) with their corresponding 95% confidence intervals (CIs). Receiver-operating characteristic curves were obtained for qualitative and quantitative CT parameters as well as hematocrit, and the corresponding areas under the curve (AUCs) were compared using the DeLong test. The Youden index method was used to identify the optimal cut-off values of the quantitative CT-derived parameter for the detection of anemia.

Cohen’s kappa (*k*) was used to evaluate intra- and inter-reader agreement for CT-derived qualitative parameters. According to *k* value agreement was graded as follows: poor (*k* value < 0.20), fair (≥ 0.20 and < 0.40), moderate (≥ 0.40 and < 0.60), good (≥ 0.60 and < 0.80), and very good (≥ 0.80 up to 1). Bland-Altman analysis was performed to compare intra- and inter-reader agreement for CT-derived quantitative parameter.

Statistical significance was set at *P*-value < .05. The Bonferroni correction was used in case of multiple comparisons, keeping statistical significance set at *P*-value < .05.

## Results

### Baseline Characteristics

The final study population consisted of 229 patients (mean age 64.5 ± 13.1 years; male, *n* = 148, BMI: 28.6 ± 20.3) who underwent both preoperative myocardial perfusion SPECT/CT and blood-test (Figure 1; Table 1). A total of 110 (48%) patients were anemic. Of those, 57 (51.8%) had mild anemia (134 < Hb ≤ 90 g/L in males, 117 < Hb ≤ 90 g/L in females), 46 (41.8%) moderate anemia (90 < Hb ≤ 60 g/L), and 7 (6.4%) severe anemia (Hb < 60 g/L). Anemic women showed lower Hb values compared to anemic men (*P* < .0001), also both BMI and hematocrit were higher in non-anemic than in anemic patients (Table 1). Age at scan and patients’ BMI had low correlation with anemia presence (*r* = − 0.04; CI − 0.17 to 0.09, and *r* = − 0.13; 95% CI − 0.25 to 0.002, respectively).

**Table 1:** Patient demographics of final study group (*n* = 229)

Characteristics	Anemia^a^ *n* = 110	No anemia *n* = 119	*P*-value
Age (year)	63.9 (14.1)	64.8 (12.3)	.5
Sex (month)	79 (71.8%)	69 (58%)	.18
Hemoglobin (g/L)*	110.5 (87.3–133.8)	141 (124.8–157.3)	
Mild anemia, *n* = 57	121.0 (116.0–127.5)	n.a.	
Moderate anemia, *n* = 46	99.5 (92.8–104.3)	n.a.	
Severe anemia, *n* = 7	71.0 (66.0–76.0)	n.a.	
Hematocrit (%)*	34 (26–42)	43 (38–48)	< .0001
BMI*	25.6 (19.4–31.8)	27.5 (17.8–37.2)	.014
Time to blood-test (day*)	1 (1–8)	2 (1–11)	.057
Surgery types			.0001
Liver surgery/transplantation	34 (30.9%)	20 (16.8%)	.09
Kidney surgery/transplantation	19 (17.3%)	4 (3.4%)	.045
Lung surgery/transplantation	18 (16.4%)	39 (32.8%)	.036
Abdominal surgery	9 (8.2%)	10 (8.4%)	1
Vascular surgery	6 (5.5%)	8 (6.7%)	1
Back surgery	4 (3.6%)	1 (0.8%)	1
Orthopedic surgery	2 (1.8%)	8 (6.7%)	.63
Bariatric surgery	1 (0.9%)	10 (8.4%)	.072
Others	17 (15.5%)	19 (16%)	1

Values are means ± standard deviations, or frequencies (percentages), unless otherwise specified

*BMI* body mass index

Moderate and severe anemia were defined as hemoglobin concentrations below 109g/l and 80g/l respectively, accordingly to the WHO criteria

*Values are expressed as median (interquartile range)

^a^Anemia was defined adopting the blood-test cutoffs routinely used at University Hospital of Zurich (hemoglobin concentrations below 135 g/L for men and below 117 g/L in women)

### Detection of Anemia - Hematocrit

Using hematocrit to detect anemia sensitivity, specificity, PPV, NPV, and AUC were 87.2%, 95.8%, 94.8%, 85.7%, and 0.893, respectively.

### Detection of Anemia - CT Qualitative Analysis

Using the standard mediastinum window settings for image visualization, the ARS was detected in 91 (39.7%) patients, the IVSS in 86 (37.6%) patients, and ARS + IVSS in 55 (24.0%) patients. When images were visualized with the modified window settings, 59 (25.8%), 98 (42.8%), and 48 (30.0%) patients were diagnosed with positive ARS, IVSS, and ARS + IVSS (Table 2). The IVSS outperformed the ARS in anemia detection, regardless of the window settings employed (Tables 3, 4). The window settings did not significantly affect the diagnostic accuracy of IVSS (*P* = .53, Table 4).

**Table 2:** Qualitative and quantitative CT findings

Sign		Anemia^a^ *n* = 110		No anemia *n* = 119	*P*-value
ARS (*n*)		63 (57.3%)		28 (23.5%)	< .001
	Confidently present	26.4%	Confidently absent	65.5%	
IVSS (*n*)		79 (71.8%)		7 (5.9%)	< .001
	Confidently present	37.3%	Confidently absent	81.5%	
ARS with modified window/level (*n*)		45 (40.9%)		14 (11.8%)	< .001
	Confidently present	13.6%	Confidently absent	74.8%	
IVSS with modified window/level (*n*)		88 (80%)		10 (8.4%)	< .001
	Confidently present	51.8%	Confidently absent	78.2%	
Septum–mean intraventricular density (HU)*		8.3 (7.1–9.9)		1.7 (0.7–2.5)	< .001

*ARS* aortic rim sign, *IVSS* inter-ventricular septum sign, *HU* Hunsfield unity

^a^Anemia was defined adopting the blood-test cutoffs routinely used at University Hospital of Zurich (hemoglobin concentrations below 135 g/L for men and below 117 g/L in women)

*Values are expressed as median (interquartile range);

**Table 3:** Accuracy measurements

	Sensitivity	Specificity	PPV	NPV	Area under ROC curve
ARS	57.3% (CI 47.5–66.7%)	76.5% (CI 67.8–83.8%)	69.2% (CI 58.7–78.5%)	65.9% (CI 57.4–73.8%)	0.669 (CI 0.609–0.729)
IVSS	71.8% (CI 62.4–80.0%)	94.1% (CI 88.3–97.6%)	91.9% (CI 83.9–96.7%)	78.3% (CI 70.7–84.8%)	0.830 (CI 0.782–0.877)
ARS with modified window/level	40.9% (CI 31.6–50.7%)	88.2% (CI 81–93.4%)	76.3% (CI 63.4–86.4%)	61.8% (CI 54.0–69.1%)	0.646 (CI 0.591–0.700)
IVSS with modified window/level	80.0% (CI 71.3–87.0%)	91.6% (CI 81.5–95.9%)	89.8% (CI 82.0–95.0%)	83.2% (CI 75.7–89.2%)	0.858 (CI 0.813–0.903)
Septum—intraventricular density	88.2% (CI 80.6–93.6%)	95.8% (CI 90.5–98.6%)	95.1% (CI 88.9–98.4%)	89.8% (CI 83.1–94.4%)	0.920 (CI 0.885–0.955)
Hematocrit	87.2% (CI 74.3–89.3%)	95.8% (CI 90.5–98.6%)	94.8% (CI 88.3–98.3%)	85.7% (CI 78.6–91.2%)	0.893 (CI 0.853–0.932)

*ARS* aortic rim sign, *IVSS* inter-ventricular septum sign, *PPV* positive predictive value, *NPV* negative predictive value, *CI* confidence interval

**Table 4:** Diagnostic performance comparisons between different qualitative methods

	IVSS	IVSS with modified window/level	ARS	ARS with modified window/level
IVSS	–	–	–	–
IVSS with modified window/level	0.353	–	–	–
ARS	< 0.001	< 0.001	–	–
ARS with modified window/level	< 0.001	< 0.001	1.000	–

*ARS* aortic rim sign, *IVSS* inter-ventricular septum sign

### Detection of Anemia-CT Quantitative Analysis

**Figure 2:**
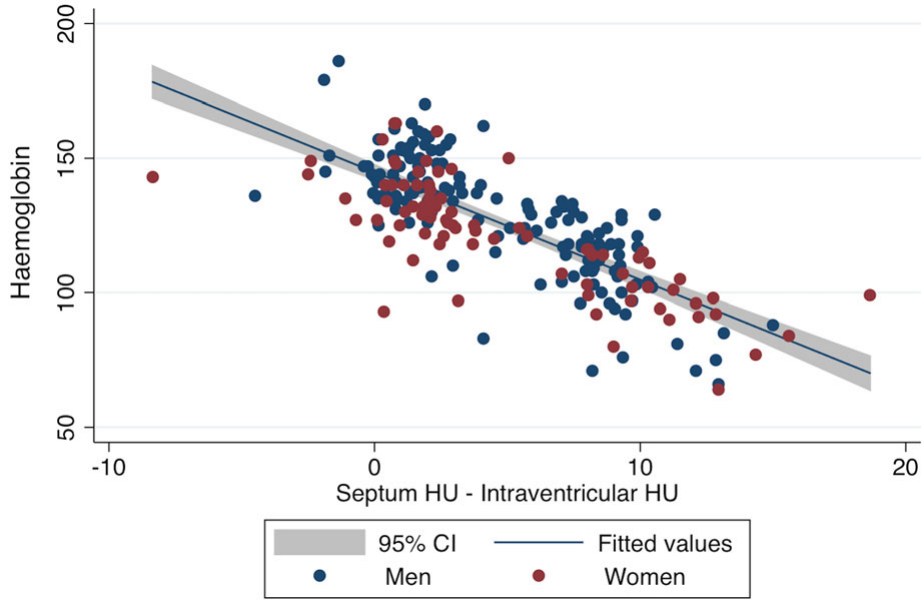
Relation between quantitative analysis and hemoglobin levels. Scatterplot graph, showing the relation between computed tomography quantitative analysis and hemoglobin levels (g/L)

**Figure 3:**
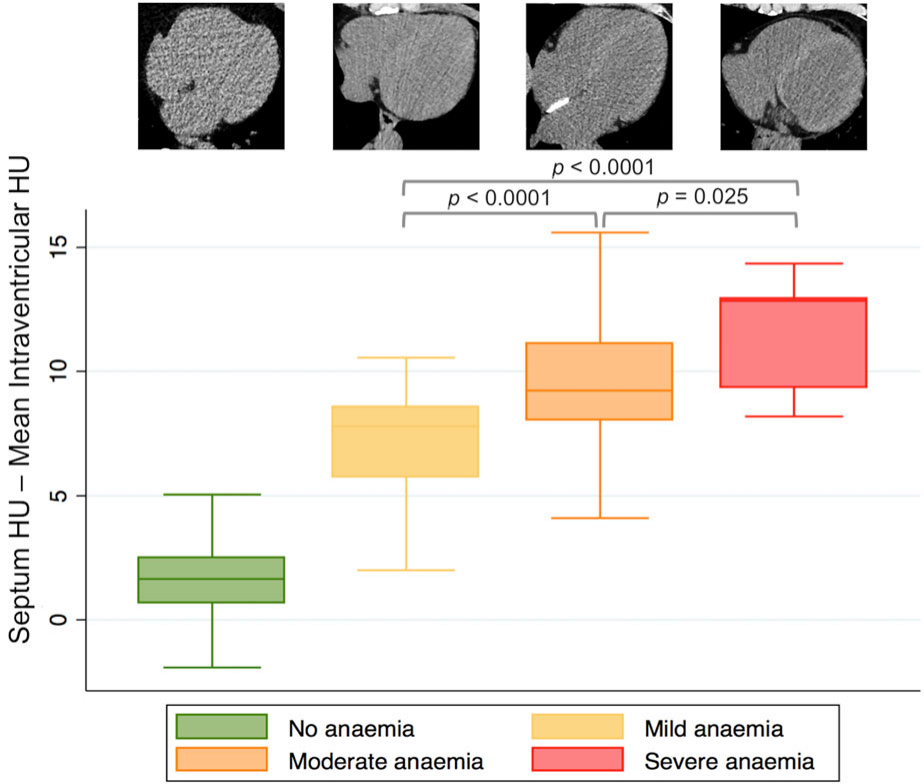
Quantitative analysis’ difference according to anemia severity. Box-plot graph showing the relation between computed tomography quantitative analysis’s differences and anemia severity. Illustrative cases overly each box-plot, illustrating the changes in blood pool density compared to the myocardium. Quantitative analysis values differed (*P *<.0001) between anemic and non-anemic, irrespective of its grade. The third illustrative case (above the orange box-plot) shows a metallic patent foramen ovale occluder located between the atria

The mean intraventricular density was significantly lower in anemic patients: 35.5 HU (32.5 to 38.1 HU) vs. 42.5 HU (40.5 to 44.7 HU; *P* < .0001), whereas IV septum density did not vary between the two groups: 43.7HU (42.1 to 45.7 HU) vs. 43.9 HU, (41.9 to 45.9 HU; *P* = .66). Although RV density values were significantly higher compared to LV ones: 40.5HU (36.0 to 43.8 HU) vs 39.1 HU (35.2 to 42.9 HU; *P* = .0005), both had a strong positive correlation with Hb values (*r*_s_ = 0.77; CIs 0.72 to 0.82 for RV, and *r*_s_ = 0.76; CIs 0.68 to 0.80 for LV). Furthermore, a strong inverse correlation between the quantitative analysis and Hb levels was found(*r*_s_ = − 0.77; CIs − 0.82 to 0.71, Figure 2). Quantitative analysis CT values were higher in anemic than in non-anemic patients: 8.4 HU (7.1 to 9.9 HU) vs 1.7 HU (0.7 to 2.5 HU; *P *< .0001). Moreover, CT values from quantitative analysis progressively increased with increasing anemia severity: 7.8 HU (5.8 to 8.6 HU), 9.3 HU (8.2 to 11.1 HU), and 12.9 HU (9.4 to 13.0 HU; *P*_mild-moderate anemia_ < .0001, *P*_mild-severe anemia_ < .0001, *P*_moderate-severe anemia_ = .025; Figure 3). Sensitivity, specificity, PPV, and NPV of the quantitative analysis were as follows: 88.2%, 95.8%, 95.1%, and 89.8% (Table 3). The optimal threshold to differentiate anemic from non-anemic patients derived using the Youden index approach was 4.5 HU. The corresponding sensitivity, specificity, and AUC were 88%, 96%, and 0.92, respectively. Similarly, sex specific cutoffs were calculated using the Youden index approach (Table 5).

**Table 5:** Quantitative values cutoffs according to sex

	HU Cutoff values	Sensitivity	Specificity	PPV	NPV	Area under ROC curve
Women	6.4	90.3% (CI 74.2–98.0%)	100% (CI 92.9–100.0%)	100% (CI 87.7–100.0%)	94.3% (CI 84.3–98.8%)	0.95 (CI 0.90–1.00)
Men	4.6	86.1% (CI 76.5–92.8%)	98.6% (CI 92.2–100.0%)	98.6% (CI 92.2–100.0%)	86.1% (CI 76.5–92.8%)	0.95 (CI 0.91–0.99)

*PPV* positive predictive value, *NPV* negative predictive value, *CI* confidence interval

### Calcified Plaque Burden of Ascending Aorta

**Figure 4:**
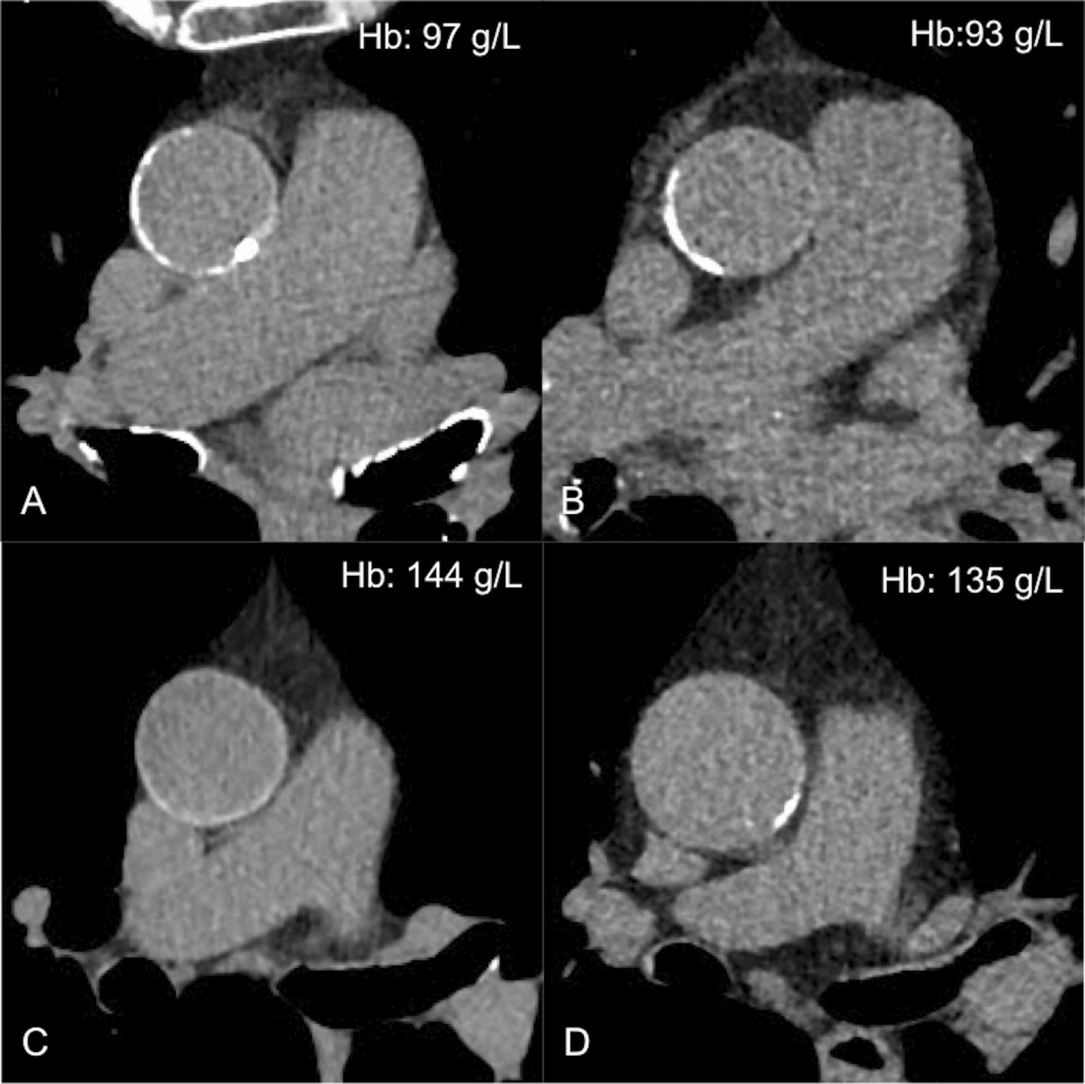
Aortic rim sign in anemic and non-anemic patients. Visibility of aortic rim sign (ARS) in four patients (two anemic, **a** and **b**, and two non-anemic, **c** and **d**), according to calcific atherosclerosis of the ascending aorta. Detecting ARS is challenging in patients with highly calcified ascending aorta (**a**, true positive case) or in those with large calcified atherosclerotic plaques (**b**, false negative case). Furthermore, circumferential aortic atherosclerosis microcalcification can mirror ARS (**c**, false positive case). On the contrary, the aortic wall and the intravascular blood are undistinguishable in negative patients (**d**, true negative case)

A total of 108 (47.2%) patients did not show calcified atherosclerotic plaques. Of the remaining 121 patients, 68 (56.2%) had spotty calcifications, whereas 53 (43.8%) had a more severe calcific plaque burden (> 10% of the circumference). Neither Hb levels nor quantitative measurements differed (*P* = .52 and *P* = .65, respectively) between these three groups. Among anemic patients with spotty calcifications the ARS was not identified in 9 patients (28%) using the standard window settings and in 13 patients (41%) using the modified window settings (Figure 4). The rate of false negative ARS increased up to 52% (standard window settings) and 66% (modified window settings) when severe aortic calcifications were detected in anemic patients (Figure 4).

### Intra- and Inter-reader Agreement for Qualitative and Quantitative CT Parameters

**Figure 5:**
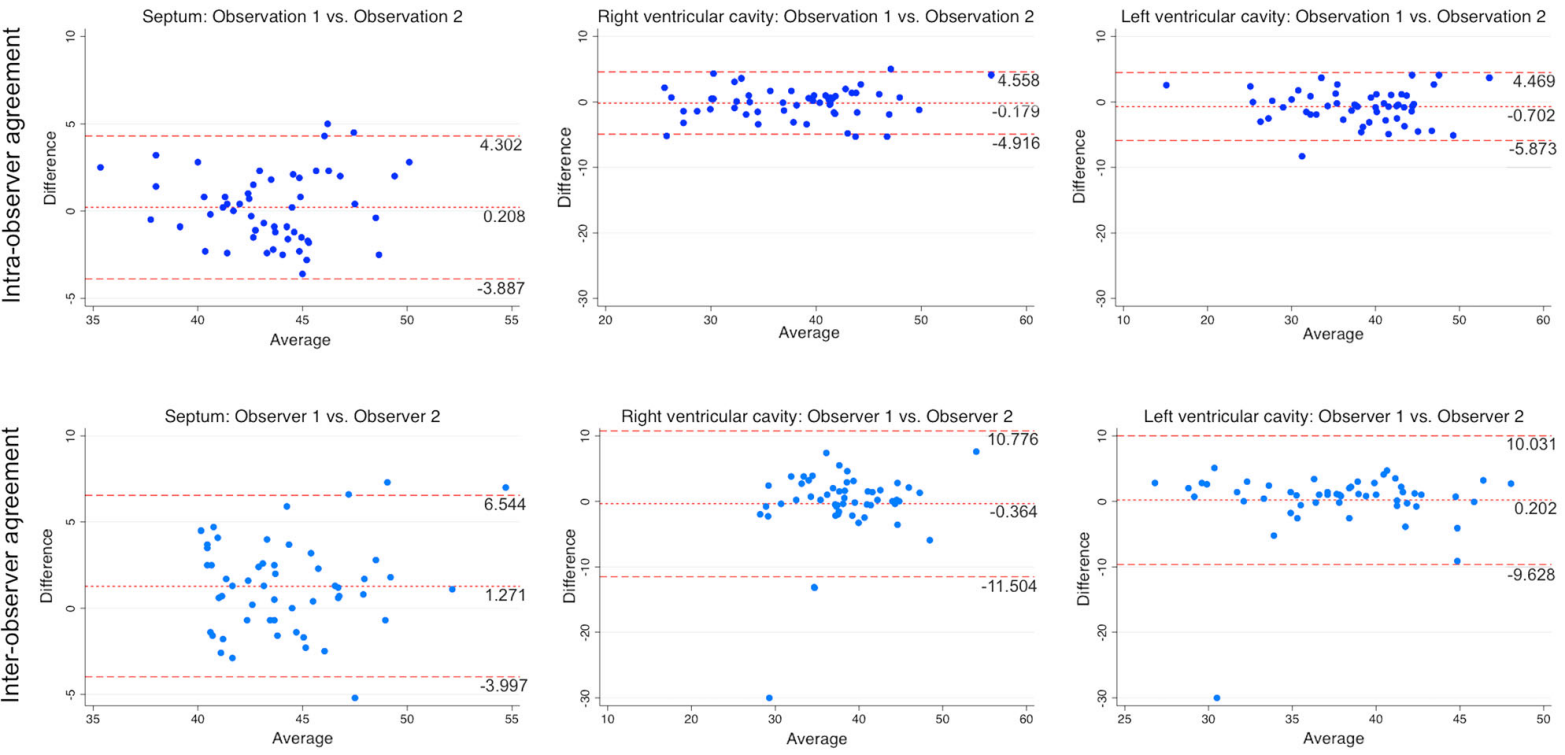
Quantitative measurements intra- and inter-reader agreement. Bland–Altman plots showing intra- (superior row) and inter-reader (inferior row) agreements between different quantitative measurements readouts. *Y*-axis represents the difference between the measurements of the two readouts. The solid line indicates the mean bias and dotted lines indicate 95% CIs

The intra-reader agreement for the detection of IVSS was very good (*k *= 0.85) with the standard window settings and very good (*k *= 0.89) with the modified window settings. The intra-reader agreement in the identification of ARS was moderate (*k *= 0.53) with the standard window settings and only fair (*k* = 0.38) with the modified window settings. On the contrary, inter-reader agreement for the detection of IVSS was good (*k *= 0.64) with the standard window, while it was very good using modified window settings (*k *= 0.93). Similarly, inter-reader agreement in the recognition of ARS was moderate (*k *= 0.58) and very good (*k *= 0.88) with standard and modified window settings, respectively. With respect to the quantitative analysis Bland–Altman plots revealed a good intra- and inter-reader agreement (Figure 5).

## Discussion

In this study, we sought to assess the diagnostic accuracy of low-dose CT images from myocardial SPECT/CT for detecting anemia comparing qualitative and quantitative approach in a population on 229 patients undergoing preoperative imaging for cardiovascular risk assessment. We observed that *inter-ventricular septum sign* was more accurate in the diagnosis of anemia than *aortic rim sign*. However, quantitative analysis outperformed qualitative analysis in the detection of anemia.

Despite anemia is often diagnosed in preoperative patients, it is still a neglected finding.^22,23^ Its incidence is three to five times greater in the elderly than in younger people below 65 years of age.^24^ Notably, in preoperative patients, anemia has been associated to increased in-hospital stay’s length and increased risk of perioperative allogeneic blood transfusion,^25^ rising the costs of health-care delivery.^25^ Therefore, the benefits associated with its early detection may help deliver improved outcomes at a lower cost.

Pre-surgical CT exams usually lack an unenhanced phase, due to radiation dose concerns. However, our study proved that unenhanced, low-dose CT images, acquired for myocardial perfusion SPECT images attenuation correction, are valuable for detecting anemia. Previous studies already reported a direct correlation between intravascular blood’s density and Hb values.^17–20^ Therefore, as Hb levels reduce, the chance to differentiate dense structures, such as the aortic wall and the IV septum, from the hypodense blood pool increases. However, these signs are subjective and related to reader’s experience. Similar to the findings reported by Kamel et al^18^ we found IVSS having higher sensitivity and specificity values compared to ARS, but our ARS sensitivity value was lower than theirs, and our intra-reader agreement evaluation of ARS, using modified window, was only fair (0.38). The differences between the two studies as well as that in the reading sessions could be attributable to the entity of aortic calcific atherosclerosis. Indeed, calcific atherosclerosis plaques in the ascending aorta likely led to ARS underestimation, as showed by the high percentages of false negatives in our study. Of note, using the modified window, also, reduced the intra-reader agreement confidence in ARS detection.

Although ARS and IVSS had good-to-high performances in anemia detection (AUCs ranging from 0.646 to 0.858) the quantitative analysis showed a higher diagnostic accuracy (AUC of 0.938). Of interest, Lan et al demonstrated that the difference between IV septum and LV intraventricular density was the most accurate parameter (AUC of 0.801).^19^ They hypothesized that subtracting the two measurements removes offset noise and image artifact increasing diagnosis accuracy.^19^

Similarly to our results, Zhou et al reported that using the quantitative analysis allowed for the discrimination between anemic and non-anemic patients.^20^ In their study they were able to differentiate severely and moderately anemic patients from non-anemic ones. Our study expanded these results showing the ability of the quantitative analysis to differentiate non-anemic patients from those with mild anemia. Interestingly, the optimal quantitative thresholds for anemia detection proposed by Zhou et al (i.e., > 5.5 HU in men, and > 4.5 HU in women) were comparable to the ones derived in our cohort.^20^ Furthermore, CT scanner parameters did not affect the ability to detect blood’s density changes. Of note, in our cohort the females quantitative threshold for anemia was higher than that of males (6.4 vs 4.6 HU). We attributed these results to the lower Hb values found in women, which generated lower intra-ventricular HU values. Interestingly, with quantitative approach we obtained a higher diagnostic accuracy than the one reported by Zhou et al^20^ This may be the result of a more precise positioning of the LV and RV ROIs. Also, in our opinion, drawing larger LV and RV ROIs (400 vs. 100 mm^2^) through the atrioventricular valves allowed a better-represented blood density, limiting the possibility to accidentally include the myocardium in the measures.

Finally, our study is the first to prove that CT quantitative analysis and hematocrit have similar diagnostic accuracy in the detection of anemia, strengthening the relationship between imaging and laboratory values. Moreover, it extends the role of myocardial SPECT-derived low-dose CT images beyond noise correction and coronary calcium burden estimation.

Our study has several limitations: First, it is a retrospective, single-center evaluation with all the inherent limitations of this study design. Second, the temporal range of Hb evaluations was wider than in other studies. However, it was reported that anemia takes longer than 29 days to develop, explaining why anesthesiologists usually suggest carrying out the pre-operative laboratory test within 30 days before surgery.^26^ Finally, although our quantitative results were comparable to those obtained in previous studies, they were derived analyzing images obtained from a single vendor CT scanner and using a single kVp, thus limiting their generalization.

## New Knowledge Gained

In preoperative patients, low-dose CT images derived from myocardial perfusion SPECT can be useful in anemia detection as well as in grading its severity. This may pave the way to expand the role of myocardial SPECT beyond that of presurgical cardiovascular risk stratification.

## Conclusion

Low-dose CT from myocardial perfusion SPECT/CT is a powerful tool to detect anemia in preoperative patients. Quantitative measurement outperformed qualitative CT assessment, having diagnostic performances comparable to blood hematocrit measurements. Based on our findings, reporting of anemia should be encouraged since it may prompt its corrections, potentially reducing patients’ morbidity.

## Supplementary Information

Below is the link to the electronic supplementary material.

Supplementary file1 (PPTX 960 kb)

Supplementary file2 (MP3 2444 kb)

## Abbreviations

WHO: World Health Organisation
Hb: Hemoglobin
SPECT: Single-photon emission computed tomography
CT: Computed tomography
ROI: Region of interest
LV: Left ventricle
RV: Right ventricle
IV septum: Inter-ventricular septum
ROC: Receiver-operating curves
IQR: Interquartile range
CI: Confidence interval
PPV: Positive predictive value
NPV: Negative predictive value
PACS: Picture Archiving and Communication system
ARS: Aortic rim sign
IVSS: Inter-ventricular septum sign
AUC: Area under ROC curve
