# Supplementary material for: Low-dose CT from myocardial perfusion SPECT/CT allows the detection of anemia in preoperative patients
Source: J Nucl Cardiol. 2022 Feb 17;29(6):3236–47. doi: 10.1007/s12350-021-02899-x (PMC9834113; doi:10.1007/s12350-021-02899-x)
Supplement: Supplementary file 1 — Supplementary file1 (PPTX 960 kb) [file 12350_2021_2899_MOESM1_ESM.pptx]

## Slide 1
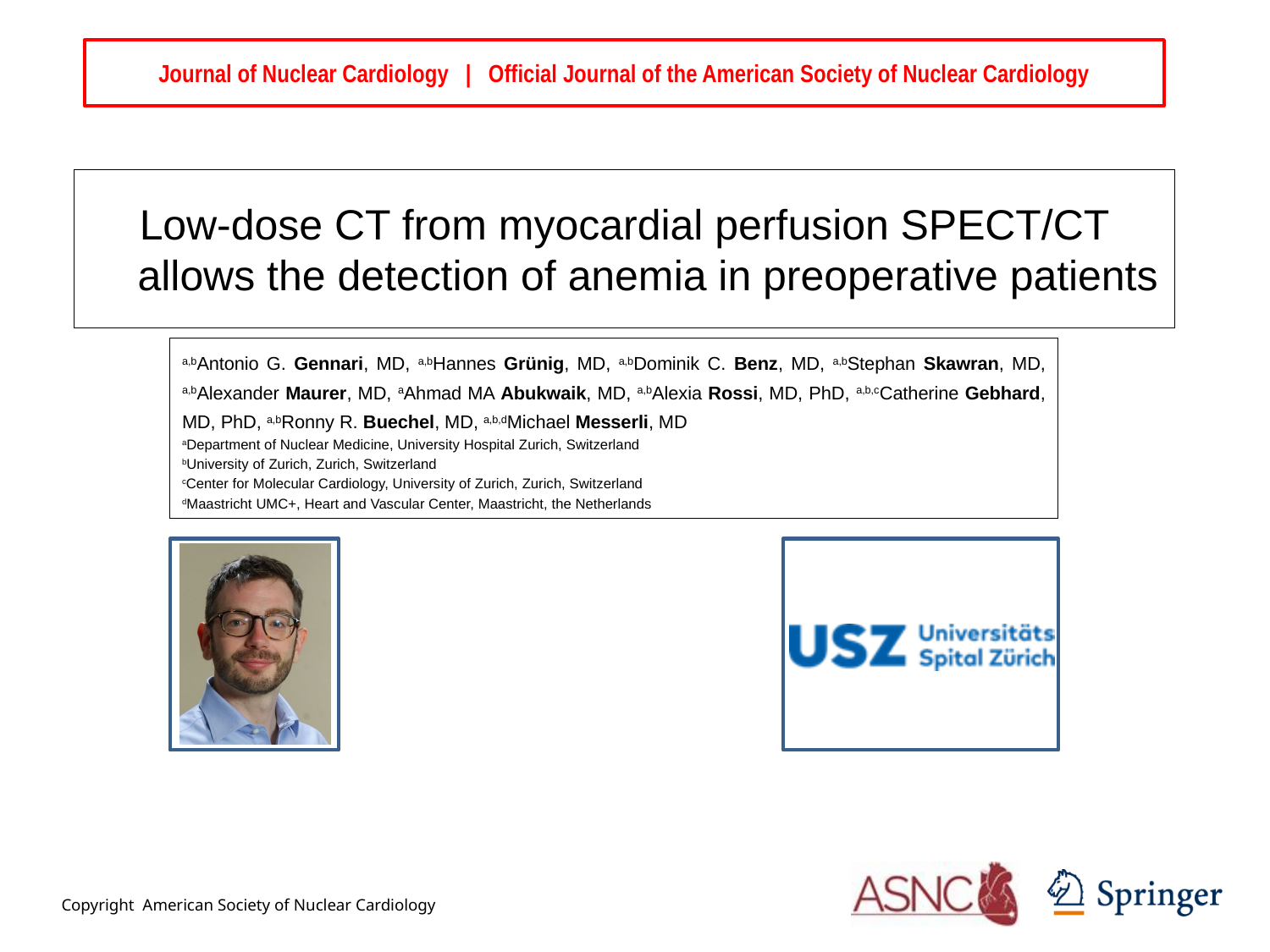

Journal of Nuclear Cardiology | Official Journal of the American Society of Nuclear Cardiology
# Low-dose CT from myocardial perfusion SPECT/CT allows the detection of anemia in preoperative patients
a,bAntonio G. Gennari, MD, a,bHannes Grünig, MD, a,bDominik C. Benz, MD, a,bStephan Skawran, MD, a,bAlexander Maurer, MD, aAhmad MA Abukwaik, MD, a,bAlexia Rossi, MD, PhD, a,b,cCatherine Gebhard, MD, PhD, a,bRonny R. Buechel, MD, a,b,dMichael Messerli, MD
aDepartment of Nuclear Medicine, University Hospital Zurich, Switzerland
bUniversity of Zurich, Zurich, Switzerland
cCenter for Molecular Cardiology, University of Zurich, Zurich, Switzerland
dMaastricht UMC+, Heart and Vascular Center, Maastricht, the Netherlands
Copyright American Society of Nuclear Cardiology

## Slide 2
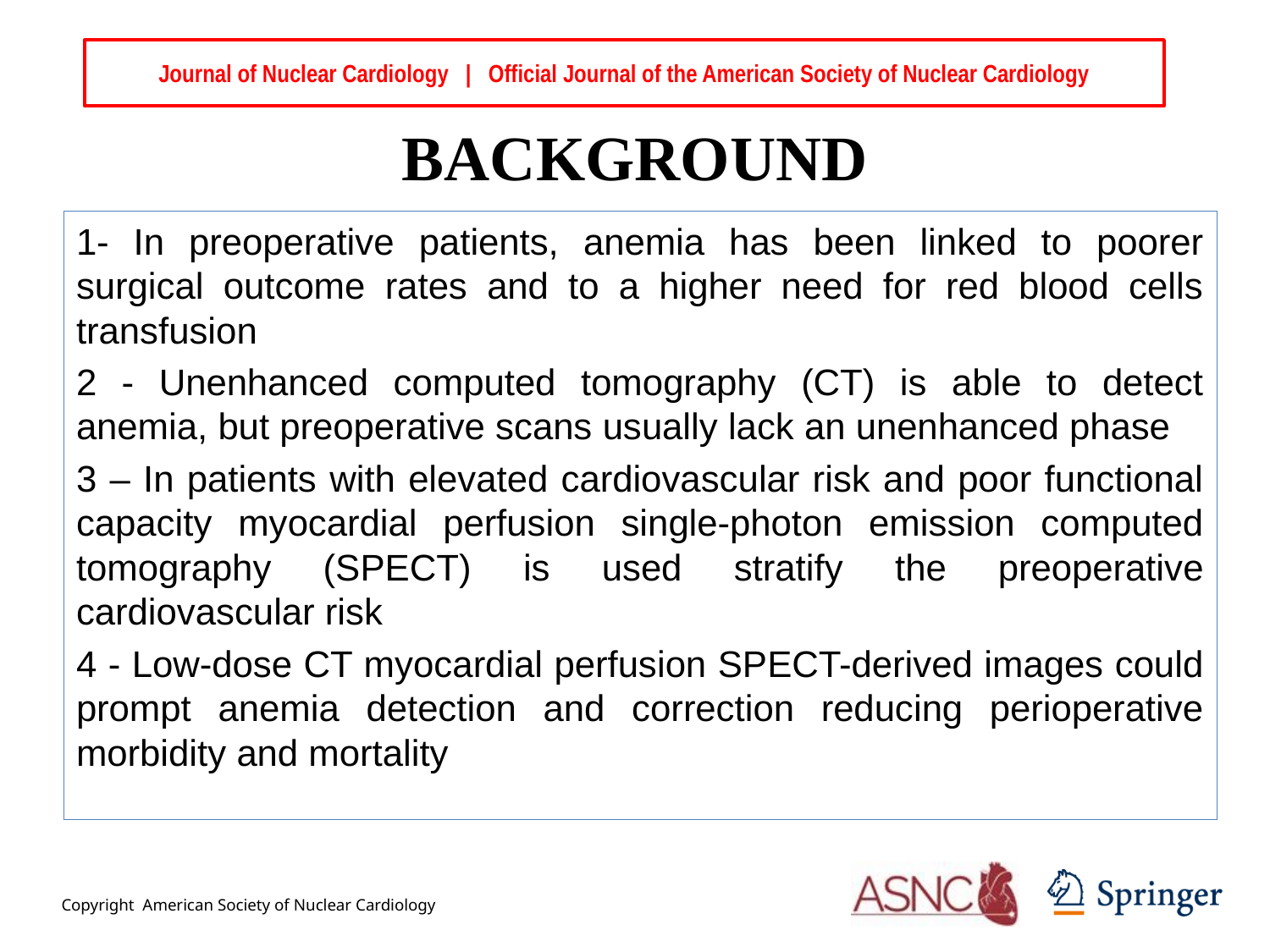

Journal of Nuclear Cardiology | Official Journal of the American Society of Nuclear Cardiology
# BACKGROUND
1- In preoperative patients, anemia has been linked to poorer surgical outcome rates and to a higher need for red blood cells transfusion
2 - Unenhanced computed tomography (CT) is able to detect anemia, but preoperative scans usually lack an unenhanced phase
3 – In patients with elevated cardiovascular risk and poor functional capacity myocardial perfusion single-photon emission computed tomography (SPECT) is used stratify the preoperative cardiovascular risk
4 - Low-dose CT myocardial perfusion SPECT-derived images could prompt anemia detection and correction reducing perioperative morbidity and mortality
Copyright American Society of Nuclear Cardiology

## Slide 3
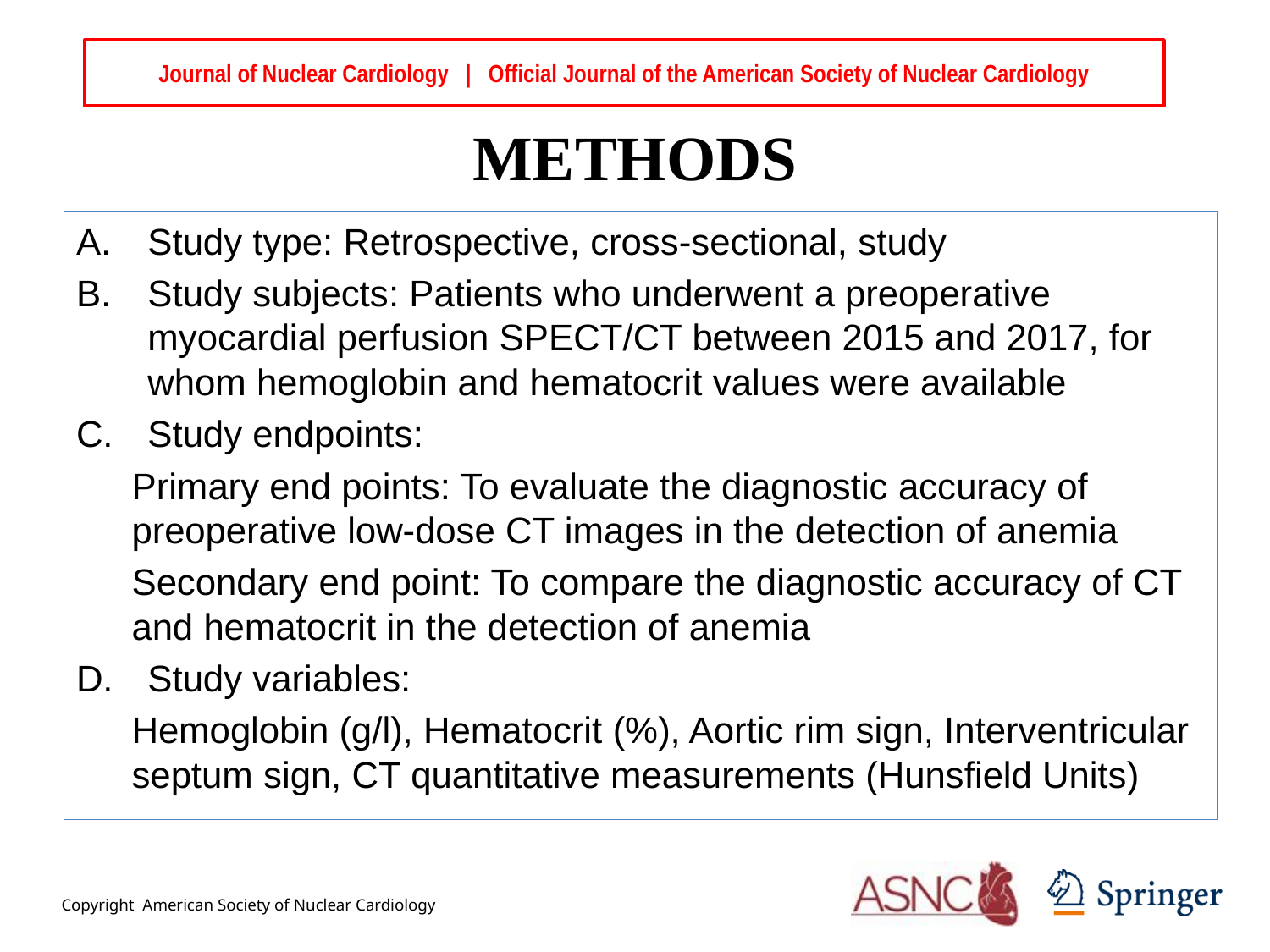

Journal of Nuclear Cardiology | Official Journal of the American Society of Nuclear Cardiology
# METHODS
Study type: Retrospective, cross-sectional, study
Study subjects: Patients who underwent a preoperative myocardial perfusion SPECT/CT between 2015 and 2017, for whom hemoglobin and hematocrit values were available
Study endpoints:
Primary end points: To evaluate the diagnostic accuracy of preoperative low-dose CT images in the detection of anemia
Secondary end point: To compare the diagnostic accuracy of CT and hematocrit in the detection of anemia
Study variables:
Hemoglobin (g/l), Hematocrit (%), Aortic rim sign, Interventricular septum sign, CT quantitative measurements (Hunsfield Units)
Copyright American Society of Nuclear Cardiology

## Slide 4
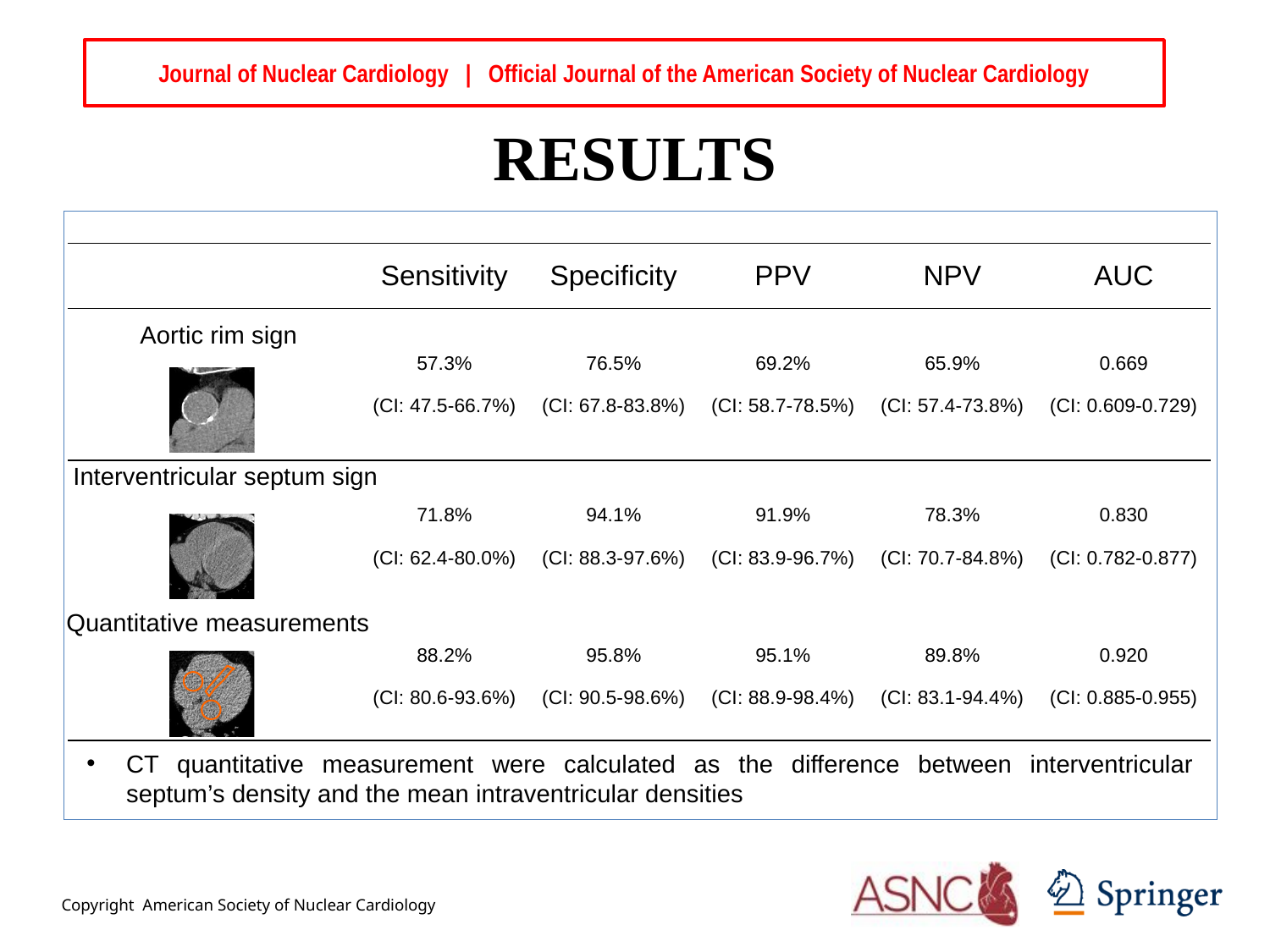

Journal of Nuclear Cardiology | Official Journal of the American Society of Nuclear Cardiology
# RESULTS
| | Sensitivity | Specificity | PPV | NPV | AUC |
| --- | --- | --- | --- | --- | --- |
| | 57.3% (CI: 47.5-66.7%) | 76.5% (CI: 67.8-83.8%) | 69.2% (CI: 58.7-78.5%) | 65.9% (CI: 57.4-73.8%) | 0.669 (CI: 0.609-0.729) |
| | 71.8% (CI: 62.4-80.0%) | 94.1% (CI: 88.3-97.6%) | 91.9% (CI: 83.9-96.7%) | 78.3% (CI: 70.7-84.8%) | 0.830 (CI: 0.782-0.877) |
| | 88.2% (CI: 80.6-93.6%) | 95.8% (CI: 90.5-98.6%) | 95.1% (CI: 88.9-98.4%) | 89.8% (CI: 83.1-94.4%) | 0.920 (CI: 0.885-0.955) |
Aortic rim sign
Interventricular septum sign
Quantitative measurements
CT quantitative measurement were calculated as the difference between interventricular septum’s density and the mean intraventricular densities
Copyright American Society of Nuclear Cardiology

## Slide 5
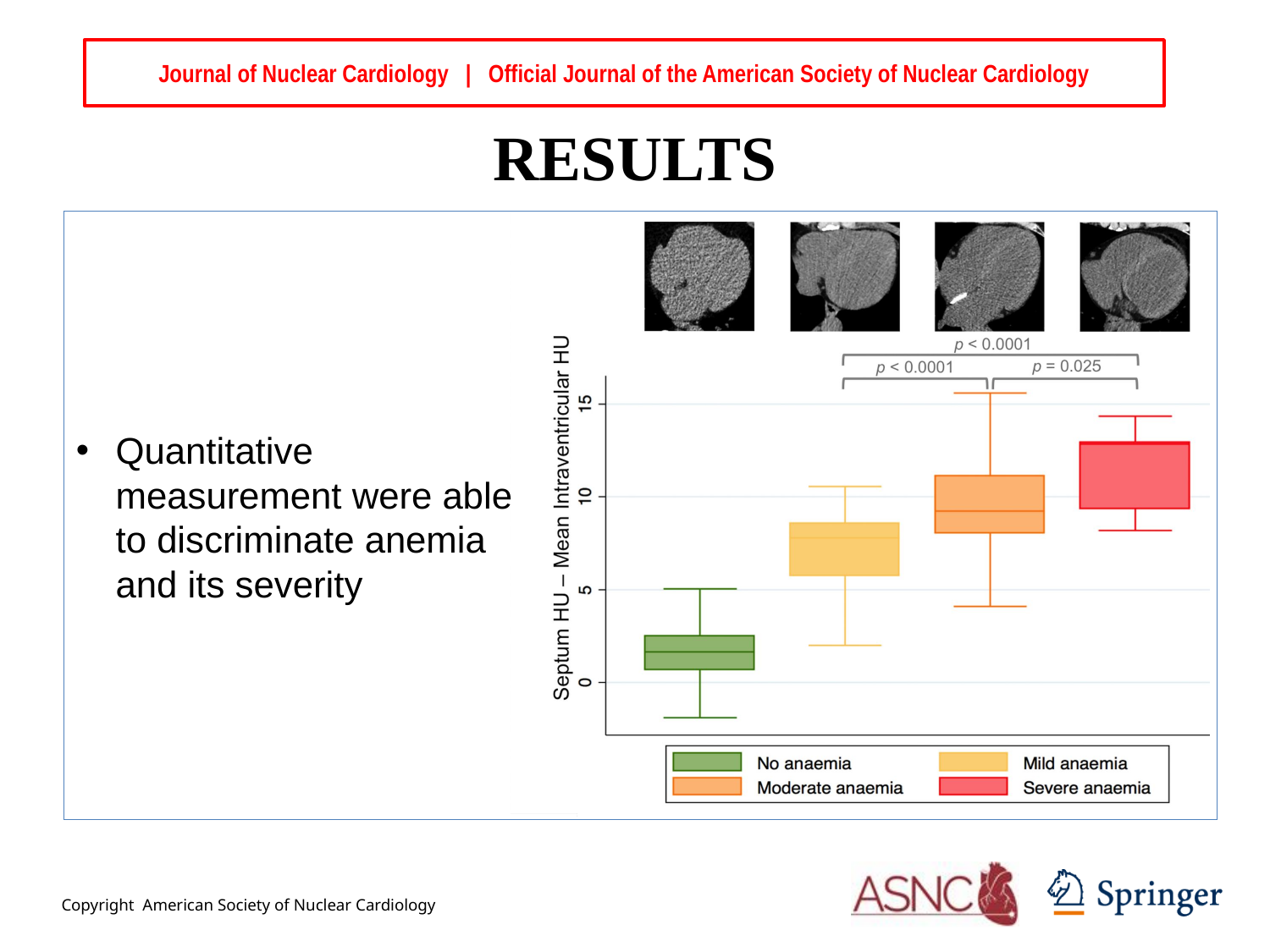

Journal of Nuclear Cardiology | Official Journal of the American Society of Nuclear Cardiology
# RESULTS
Quantitative measurement were able to discriminate anemia and its severity
Copyright American Society of Nuclear Cardiology

## Slide 6
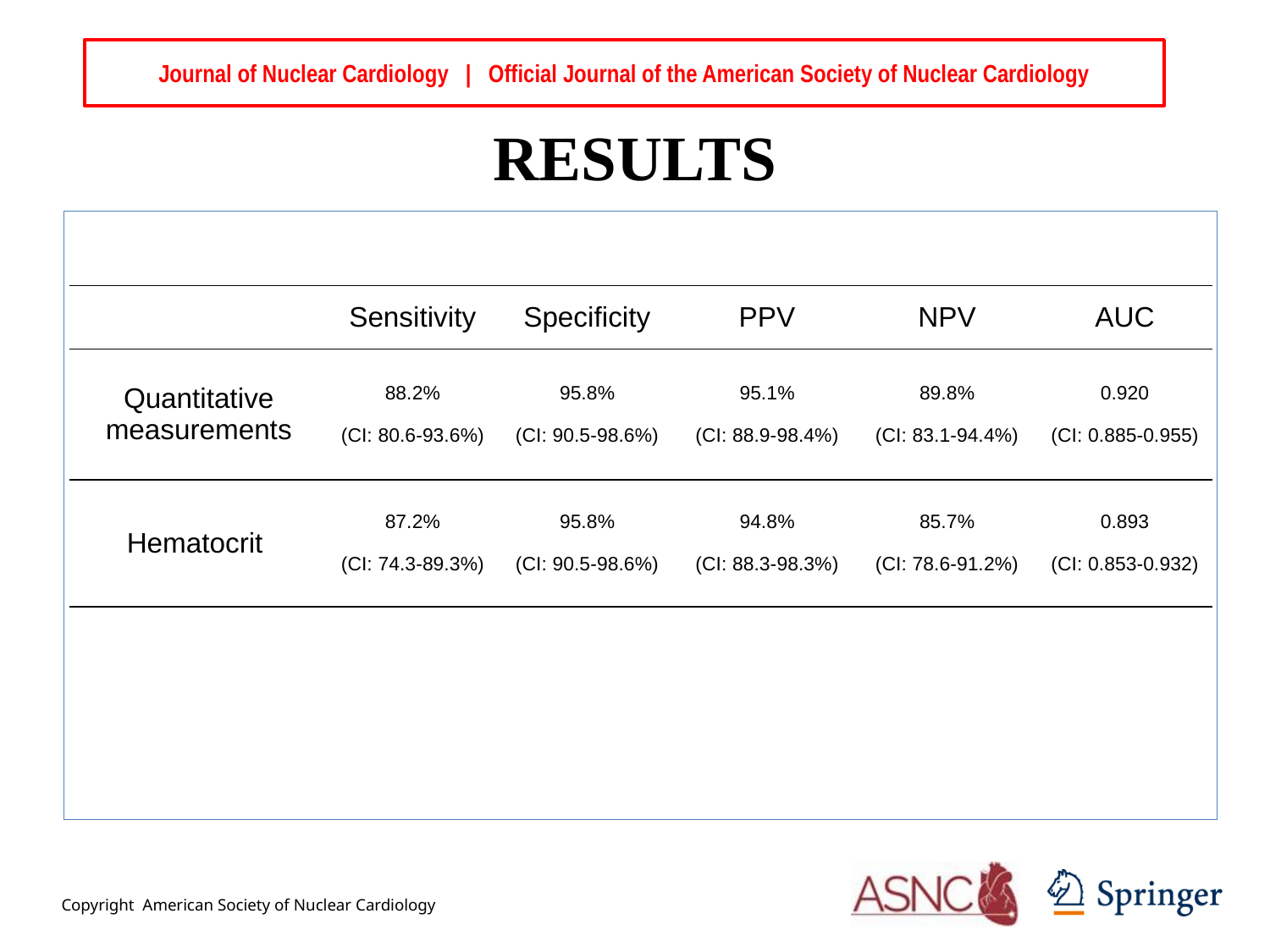

Journal of Nuclear Cardiology | Official Journal of the American Society of Nuclear Cardiology
# RESULTS
| | Sensitivity | Specificity | PPV | NPV | AUC |
| --- | --- | --- | --- | --- | --- |
| Quantitative measurements | 88.2% (CI: 80.6-93.6%) | 95.8% (CI: 90.5-98.6%) | 95.1% (CI: 88.9-98.4%) | 89.8% (CI: 83.1-94.4%) | 0.920 (CI: 0.885-0.955) |
| Hematocrit | 87.2% (CI: 74.3-89.3%) | 95.8% (CI: 90.5-98.6%) | 94.8% (CI: 88.3-98.3%) | 85.7% (CI: 78.6-91.2%) | 0.893 (CI: 0.853-0.932) |
Copyright American Society of Nuclear Cardiology

## Slide 7
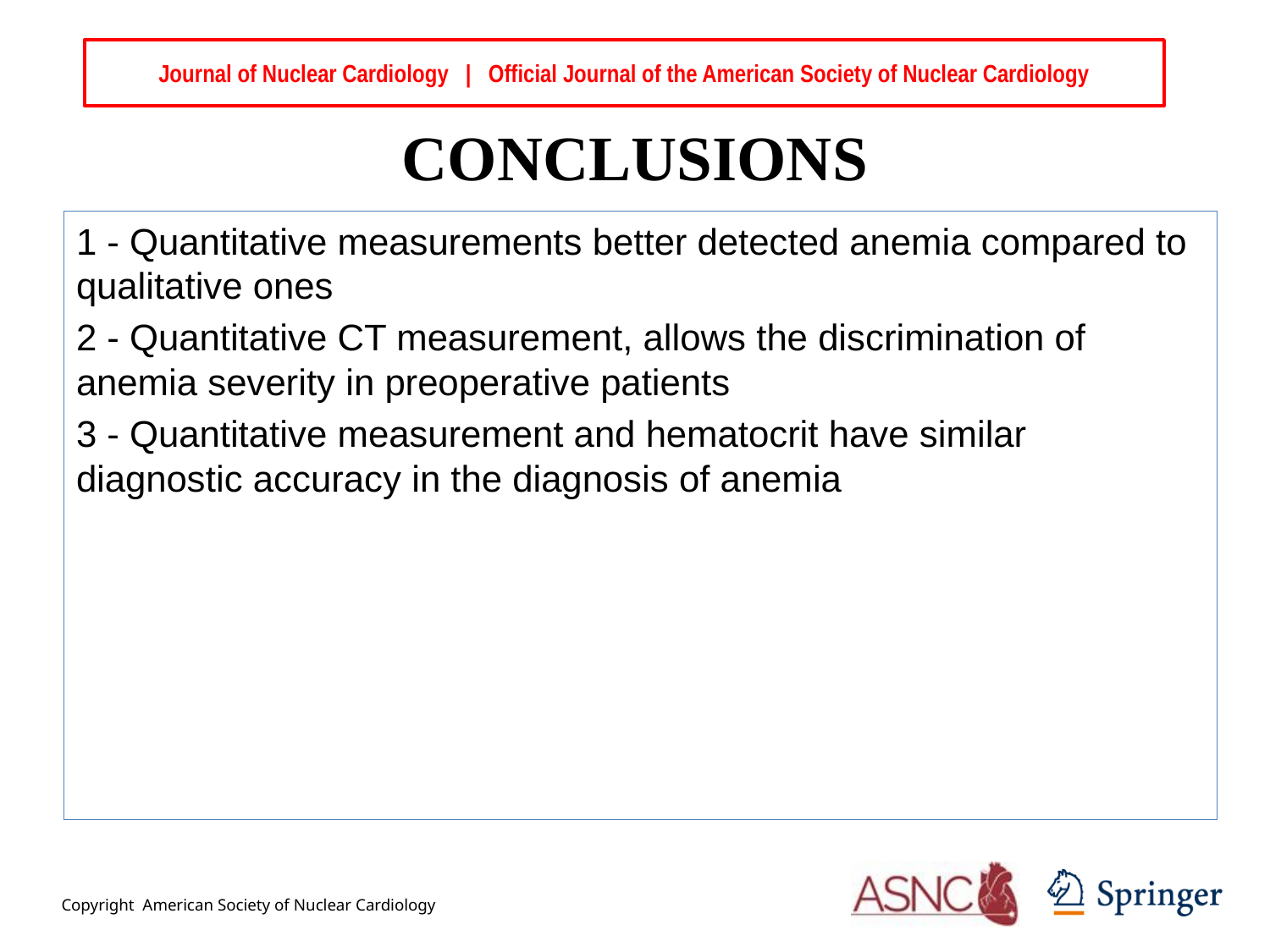

Journal of Nuclear Cardiology | Official Journal of the American Society of Nuclear Cardiology
# CONCLUSIONS
1 - Quantitative measurements better detected anemia compared to qualitative ones
2 - Quantitative CT measurement, allows the discrimination of anemia severity in preoperative patients
3 - Quantitative measurement and hematocrit have similar diagnostic accuracy in the diagnosis of anemia
Copyright American Society of Nuclear Cardiology
